# Estimating and visualising multivariable Mendelian randomization analyses within a radial framework

**DOI:** 10.1371/journal.pgen.1011506

**Published:** 2024-12-16

**Authors:** Wes Spiller, Jack Bowden, Eleanor Sanderson

**Affiliations:** 1 Population Health Sciences, University of Bristol, Bristol, United Kingdom; 2 MRC Integrative Epidemiology Unit, University of Bristol, Bristol, United Kingdom; 3 University of Exeter Medical School, Exeter, United Kingdom; 4 Novo Nordisk Genetics Centre of Excellence, Oxford, United Kingdom; The University of Chicago, UNITED STATES OF AMERICA

## Abstract

**Background:**

Mendelian randomization (MR) is a statistical approach using genetic variants as instrumental variables to estimate causal effects of a single exposure on an outcome. Multivariable MR (MVMR) extends this to estimate the direct effect of multiple exposures simulatiously. MR and MVMR can be biased by the presence of pleiotropic genetic variants in the set used as instrumental variables, violating one of the core IV assumptions. Genetic variants that give outlying estimates are often considered to be potentially pleiotropic variants. Radial plots can be used in MR to help identify these variants. Analogous plots for MVMR have so far been unavailable due to the multidimensional nature of the analysis.

**Methods:**

We propose a radial formulation of MVMR, and an adapted Galbraith radial plot, which allows for the estimated effect of each exposure within an MVMR analysis to be visualised. Radial MVMR additionally includes an option for removal of outlying SNPs which may violate one or more assumptions of MVMR. A RMVMR R package is presented as accompanying software for implementing the methods described.

**Results:**

We demonstrate the effectiveness of the radial MVMR approach through simulations and applied analyses. We highlight how outliers with respect to all exposures can be visualised and removed through Radial MVMR. We present simulations that illustrate how outlier removal decreases the bias in estimated effects under various forms of pleiotropy. We apply Radial MVMR to estimate the effect of lipid fractions on coronary heart disease (CHD). In combination with simulated examples, we highlight how important features of MVMR analyses can be explored using a range of tools incorporated within the RMVMR R package.

**Conclusions:**

Radial MVMR effectively visualises causal effect estimates, and provides valuable diagnostic information with respect to the underlying assumptions of MVMR.

## Author summary

Mendelian Randomisation (MR) can be used to estimate whether a potential exposure has a causal effect on an outcome in the presence of a third (unobserved) variable that affects both of them and so biases the observed association between them. This can be extended to estimate the direct effect of multiple exposures on a single exposure through multivariable MR (MVMR). MR and MVMR can be biased by the presence of pleiotropic genetic variants in the set used as instrumental variables, violating one of the core IV assumptions. Genetic variants that give outlying estimates are often considered to be potentially pleiotropic variants. Radial plots can be used in MR to help identify these variants. In this paper we present a novel Radial MVMR. Radial MVMR can identify outlying, potentially pleiotropic variants in MVMR and enable estimation of the causal effects with these variants excluded. Radial MVMR has the added benefit of enabling visualisation of these variants and the causal effect estimates obtained through plots which is not possible with standard plots due to the multidimensional nature of MVMR models. Alongside the description and illustration of the method we provide an R package to enable implementation of Radial MVMR.

## Introduction

Mendelian randomization (MR) is a methodological framework in which genetic variants- often single nucleotide polymorphisms (SNPs)- are used as instrumental variables to estimate causal effects in the presence of unmeasured confounding [1, 2]. MR analyses are often performed using summary data from publicly available genome-wide association studies (GWAS), reflecting the ease with which such data can be accessed in contrast with individual-level data [3].

The extent to which MR causal effect estimates are unbiased is largely determined by three key assumptions. SNPs serving as instruments must be robustly associated with the exposure of interest (IV1), there must be no confounding of the SNPs and the outcome (IV2) and there must be no effect of the SNPs on the outcome that does not act via the exposure, i.e. no horizontal pleiotropy (IV3) [4]. If all of these assumptions are satisfied for every SNP then the causal effect estimated by each SNP should differ only due to statistical chance. Heterogeneity in the per-SNP causal effects estimates is often taken to be an indication of violation of the IV assumptions and potential evidence of pleiotropic effects biasing the estimates obtained for some SNPs. SNPs that contribute to this heterogeneity by more than would be expected by chance are often considered to be outliers and their role in any potential pleiotropic effects investigated further. [5, 6] Radial MR has previously been proposed as a method for MR that allows for visualisation and removal of outliers, as measured by the SNPs that contribute more than expected by chance to heterogeneity in the analysis [7].

Multivariable MR (MVMR) is an extension to standard MR estimation which allows for the inclusion of multiple exposures, estimating the direct effect of each exposure on the outcome of interest, conditional on the other exposures included in the model [8–10]. MVMR can be used to obtain pleiotropy robust effect estimates when the mechanism by which the pleiotropy is acting is understood and measured. However, often it is unclear what the mechanism by which pleiotropic effects are acting, or many potential effects are thought to be present. A range of pleiotropy robust methods have been proposed for MVMR [11–13], however these do not focus on the identification of the SNPs contributing to heterogenetiy in MVMR.

The multidimensional nature of MVMR means that it cannot be visualised in scatter plots as simply as MR. This makes visual inspection of outliers, or comparison of effects challenging. In this paper we present a Radial MVMR approach which allows for visualisation of the effects obtained in MVMR and identification of SNPs that are outlying based on their association with all of the exposures. We then show how Radial MVMR can be used to prune outliers and so obtain results that are not influenced by outlying SNPs. We illustrate the method through a simulation across a range of levels of balanced and unbalanced pleiotropy.

To demonstrate the application of Radial MVMR we present an applied example evaluating the effects of low-density lipoprotein (LDL), high-density lipoprotein (HDL), and triglycerides on coronary heart disease (CHD). We illustrate how univariable MR estimates for each of the exposures are biased by pleiotroic effects via the other exposures considered. We then show how the application of Radial MVMR can be used to visualise the MVMR results and to obtain more robust effect estimates through the identification and removal of SNPs which contribute more to heterogenetiy in the MVMR results than would be expected by chance. Throughout we perform all analyses using the RMVMR R package for the R software environment, which has been developed to facilitate the application of radial MVMR analyses. The RMVMR R package is available from https://github.com/WSpiller/RMVMR.

## Methods

### Univariable summary Mendelian randomization

**Fig 1:**
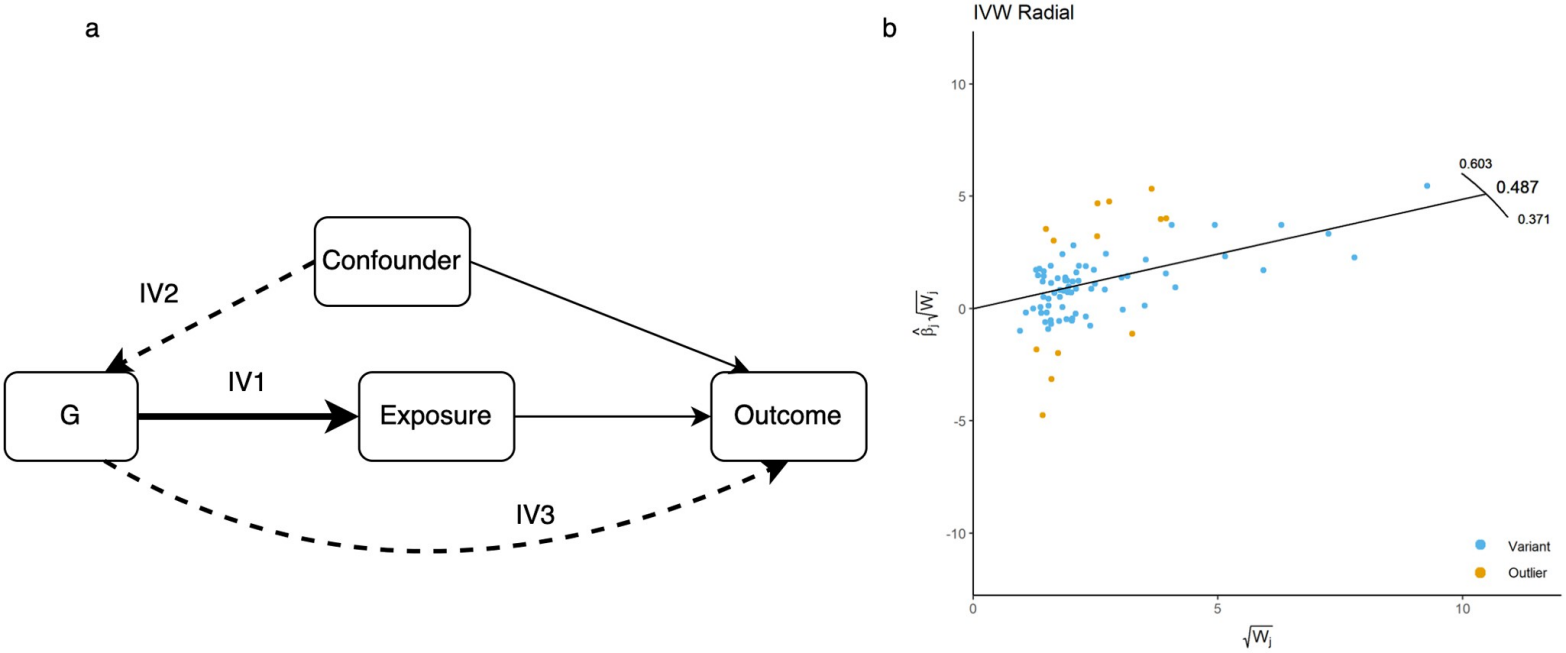
Directed acyclic graph (DAG) illustrating the assumptions of MR and an illustration of a Radial MR plot. a. Illustrates the IV assumptions required for MR, the dashed line shows paths that are assumed not to exist for *G* to be a valid instrument. b. A Radial MR plot with the outlying SNPs highlighted in orange.

In univariable MR analyses one or more SNPs are used as instruments to estimate the causal effect of a single exposure *X* upon an outcome *Y*. Let *G*_*j*_ represent the *j*^*th*^ independent SNP from a set of *j* ∈ {1, 2, …, *J*}, and let *U* denote one or more unmeasured confounders. A SNP is considered valid provided it satisfies assumptions IV1–3, with assumed relationships depicted in Fig 1.

SNP-exposure and SNP-outcome associations are obtained from GWAS results that have been adjusted for population structure. Defining ${\hat{\gamma }}_{j}$ as the association between SNP *j* and the exposure *X* obtained from a GWAS of the exposure and ${\hat{\Gamma }}_{j}$ as the association between SNP *j* and the outcome *Y* obtained from a GWAS of the outcome, the per-SNP MR estimate of the effect of *X* on *Y* is given by;

$$\begin{array}{c} {\hat{\beta }}_{j}=\frac{{\hat{\Gamma }}_{j}}{{\hat{\gamma }}_{j}} \end{array}$$ (1)

Combining this across *j* = (1, …, *J*) uncorrelated SNPS the Inverse Variance Weighted (IVW) MR estimate is given by;

$$\begin{array}{c} {\hat{\beta }}_{IVW}=\frac{\sum_{j=1}^{J}w_{j}{\hat{\beta }}_{j}}{\sum_{j=1}^{J}w_{j}}, \end{array}$$ (2)

Where $w_{j}={\hat{\gamma }}_{j}^{2}/{\hat{\sigma }}_{Yj}^{2}$ is the weighting applied to each SNP, ${\hat{\sigma }}_{Yj}^{2}$ is the variance of the estimated SNP-outcome association ${\hat{\Gamma }}_{j}$. [3] As described in Bowden et al (2018), an equivalent IVW estimate in Eq 2 can be obtained by fitting a radial regression model, regressing the product of the ratio estimate and square root weight attributed to each SNP against the set of square root weights across all SNPs [7].

$$\begin{array}{c} {\hat{\beta }}_{j}\sqrt{w_{j}}=\beta_{IVW}\sqrt{w_{j}}+\epsilon_{j} \end{array}$$ (3)

A key advantage of this transformation is that *ϵ*_*j*_ in this regression, i.e. the distance of each observation from the fitted regression line is equal to its square-root contribution to Cochran’s heterogeneity statistic, $\sqrt{Q_{j}}$, where

$$\begin{array}{c} Q=\sum Q_{j}=\sum_{j=1}^{J}w_{j}{\left(\beta_{j}-\beta_{IVW}\right)}^{2} \end{array}$$ (4)

Therefore by plotting ${\hat{\beta }}_{j}\sqrt{w_{j}}$ against $\sqrt{w_{j}}$ it is possible to visualise the heterogeneity across SNPs in the data. The SNPs that influence this heterogeneity most, i.e. those with the largest values of *w*_*j*_(*β*_*j*_ − *β*_*IVW*_)^2^, can then be identified and pruned to obtain effect estimates that are not influenced by outlying values. This is most commonly done by removing all SNPs that explain more variation in *Q* than would be expected by chance and so have an individual SNP *Q*-statistic, *Q*_*j*_ that has a p-value of less than 0.05.

### Multivariable Mendelian randomization

MVMR extends the univariable MR framework to include multiple potentially correlated exposures, leveraging the entire set of SNPs associated with at least one included exposure [8, 10]. This allows for the direct effect of each exposure to be consistently estimated, (that is, the effect of an exposure holding the others fixed), provided that the MVMR assumptions hold;

- SNPs serving as instruments must be robustly associated each exposure conditional on the other exposures included in the model (MV-IV1)
- There is no confounding of the SNPs and the outcome (MV-IV2)
- There is no effect of the SNPs on the outcome that does not act via at least one of the exposures in the model (MV-IV3)

**Fig 2:**
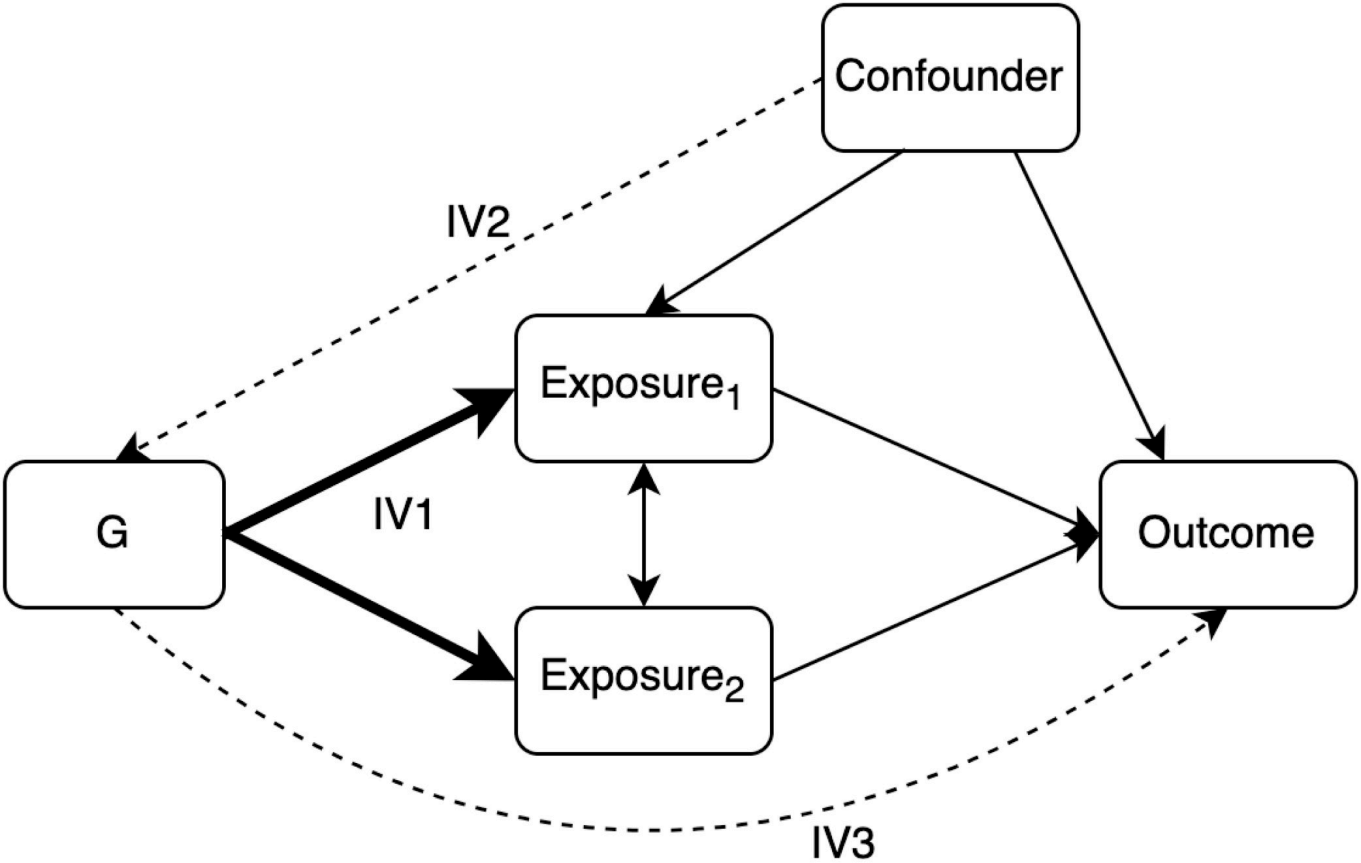
A DAG illustrating the assumptions required for MVMR. Dashed lines represent associations which must not exist for *G* to represent a set of valid instruments for Exposure 1 and Exposure 2.

These assumption are illustrated in Fig 2.

For *i* = {1, 2, …, *N*} observations including *j* = {1, 2, …, *J*} SNPs and *k* = {1, 2, …, *K*} exposures, defining *m* = {1, 2, …, *M*} as the exposures included in the model other than *k*, for which *k* ∉ *m*, a data generating process can be described as;

$$\begin{array}{c} X_{ki}=\gamma_{k0}+\sum_{j=1}^{J}\gamma_{kj}G_{ji}+\gamma_{k(J+1)}U_{i}+\sum_{m=1}^{M}\delta_{km}X_{mi}+\epsilon_{Xki} \end{array}$$ (5)

$$\begin{array}{c} Y_{i}=\beta_{0}+\sum_{k=1}^{K}\beta_{k}X_{ki}+\sum_{j=1}^{J}\alpha_{j}G_{ji}+\beta_{\left(K+1\right)}U_{i}+\epsilon_{Yi} \end{array}$$ (6)

Where *U* is an unobserved confounder and *α*_*j*_ is the pleiotropic effect of SNP *j* on the outcome which is assumed to be zero for the MVMR assumptions to be satisfied. Using Eqs (5) and (6) the univariable estimand for a single exposure can be derived. For clarity, we denote the total effect of an instrument *G*_*j*_ on an exposure *X*_*k*_ as $\gamma_{kj}^{*}$, while *γ*_*kj*_ represents the direct effect of *G*_*j*_ on *X*_*k*_ conditioning on all relevant exposures on the pathway from *G*_*j*_ to *X*_*k*_. This allows us to define the univariable ratio estimand as:

$$\begin{array}{c} \gamma_{kj}^{*}=\gamma_{kj}+\sum_{m=1}^{M}\delta_{km}\gamma_{mj} \end{array}$$ (7)

$$\begin{array}{c} \Gamma_{j}=\sum_{k=1}^{K}\beta_{k}\gamma_{kj}^{*}+\alpha_{j}\;\; \end{array}$$ (8)

$$\begin{array}{c} \frac{\Gamma_{j}}{\gamma_{kj}^{*}}=\beta_{k}+(\frac{\sum_{m=1}^{M}\beta_{m}\gamma_{mj}^{*}+\alpha_{j}}{\gamma_{kj}+\sum_{m=1}^{M}\delta_{km}\gamma_{mj}^{*}}) \end{array}$$ (9)

If the MVMR assumptions are satisfied, and independent SNPs are used, Eq (9) simplifies to

$$\begin{array}{c} \frac{\Gamma_{j}}{\gamma_{kj}^{*}}=\beta_{k}+(\frac{\sum_{m=1}^{M}\beta_{m}\gamma_{mj}^{*}}{\gamma_{kj}^{*}}) \end{array}$$ (10)

Note that the term in parentheses in Eq 10 represents the effect of the additional exposures *X*_*m*_, and including ratio estimates for all additional exposures *X*_*m*_ within a multivariable regression will yield marginal effects of each exposure, adjusting for this term in each case. In univariable MR, pleiotropic effects violating IV3 would likely be present when $\beta_{m}{\hat{\gamma }}_{mj}\neq 0$. For a MVMR with *k* exposures, the direct effect of each exposure can be estimated by regressing instrument-outcome associations upon instrument-exposure associations for each exposure simultaneously [3], such that:

$$\begin{array}{c} {\hat{\Gamma }}_{j}=\beta_{IVW1}{\hat{\gamma }}_{1j}+\beta_{IVW2}{\hat{\gamma }}_{2j}+\ldots +\beta_{IVWk}{\hat{\gamma }}_{kj}+\epsilon_{j} \end{array}$$ (11)

Where ${\hat{\gamma }}_{kj}$ is the estimated association between SNP *j* and exposure *k*.

### Extending the radial framework to MVMR

The univariable radial MR model can be readily extended to include multiple exposures, creating an analogue of the MVMR regression model shown in Eq 12:

$$\begin{array}{c} {\hat{\beta }}_{1j}\left(\sqrt{w_{1j}}\right)=\beta_{IVW1}\left(\sqrt{w_{1j}}\right))+\beta_{IVW2}\sqrt{w_{2j}}+\ldots +\beta_{IVWK}\sqrt{w_{Kj}}+\epsilon_{j} \end{array}$$ (12)

where *w*_*kj*_ represents the weighting for each SNP with respect to exposure *k*. This weighting is given by;

$$\begin{array}{c} w_{kj}=\frac{{\hat{\gamma }}_{k,j}}{{\hat{\sigma }}_{Yj}^{2}} \end{array}$$ (13)

Where ${\hat{\gamma }}_{kj}$ is defined as before and ${\hat{\sigma }}_{Yj}^{2}$ is the estimated variance of the association between the outcome *Y* and SNP *j*.

An immediate benefit of conducting MVMR within a radial framework is plotting ${\hat{\beta }}_{kj}\sqrt{w_{kj}}$ against $\sqrt{w_{kj}}$ can be accomplished using generalised axes scales, allowing ratio estimates for each exposure to be projected onto the same scatter plot simultaneously. The RMVMR plot has the same x-axis scale as the univariable Radial MR plot, plotting $\sqrt{w_{j}}$ values. The y-axis of the RMVMR plot, however, is the product of the ratio estimate and weighting for the reference exposure. As all instruments associated with at least one exposure are used to estimate causal effects for every exposure, an RMVMR plot will have *K* × *J* observations. i.e. the plot will include a set of weightings for all SNPs for each exposure. While all weightings are used to estimate causal effects it may be appropriate to limit the number of observations represented on an RMVMR plot to instruments exceeding a given weighting threshold. For example, points corresponding to an exposure *X*_1_ would be shown on the RMVMR plot provided they have an F-statistic greater than 10 for *X*_1_. This omits clusters of instruments with negligible weightings which are unlikely to be of interest, while improving the readability of the plot.

RMVMR plots are particularly useful as a tool for highlighting the extent to which individual SNPs contribute towards global heterogeneity with respect to each included exposure. Performing a univariable MR analyses will result in biased estimates where SNPs are associated with multiple causally relevant phenotypes. This is primarily because the sum of associations $\sum_{m=1}^{M}\beta_{m}{\hat{\gamma }}_{mj}$ represent pleiotropic pathways through the omitted exposures, resulting in increased heterogeneity provided such effects are not identically distributed across the set of SNPs. If this is the case, then it follows that adjusting for such associations would result in a decrease in effect estimate heterogeneity, with estimates converging towards the MVMR estimate once the univariable pleiotropic bias is corrected. The radial analogue for Eq (10) can be written as:

$$\begin{array}{c} \frac{\Gamma_{j}}{\sqrt{w_{kj}}}=\beta_{k}+(\frac{\sum_{m=1}^{M}\beta_{m}\sqrt{w_{mj}}}{\sqrt{w_{kj}}}) \end{array}$$ (14)

This result has important implications in terms of visualising heterogeneity in RMVMR analyses. In an RMVMR plot we plot the product of the ratio estimate and corresponding square root weighting against each set of weights on a generalised x-axis ($\sqrt{w_{j}}$). However, using the univariable ratio estimate for each instrument, superimposed regression lines representing the RMVMR estimate for each exposure will not represent the best fit through the plotted observations. This is because the ratio estimates do not account for the adjustment from other exposures. We can write the position of each data point on the y-axis as:

$$\begin{array}{cc} \beta_{kj}\sqrt{w_{kj}} & =\beta_{k}\sqrt{w_{kj}}+(\frac{\sum_{m=1}^{M}\beta_{m}\sqrt{w_{mj}}}{\sqrt{w_{kj}}})\sqrt{w_{kj}}\\ & =\beta_{k}\sqrt{w_{kj}}+\sum_{m=1}^{M}\beta_{m}\sqrt{w_{mj}} \end{array}$$ (15)

Therefore, by subtracting $\sum_{m=1}^{M}\beta_{m}\sqrt{w_{mj}}$ from the y-axis value of each data point, an adjustment can be performed to account for the other exposures included in the RMVMR model. Crucially, though the true value of *β*_*m*_ is unknown in Eq 15, this adjustment can be made using the estimate of *β*_*m*_ obtained from the RMVMR model, that is, *β*_*IVWm*_ in Eq 12. From this we can develop an adapted form of Cochran’s Q-statistic calculated for each exposure in a Radial MVMR model:

$$\begin{array}{c} Q_{k}=\sum_{j=1}^{J}Q_{kj}=\sum_{j=1}^{J}w_{kj}{\left(\beta_{kj}-(\frac{\sum_{m=1}^{M}\beta_{m}\sqrt{w_{mj}}}{\sqrt{w_{kj}}})-\beta_{IVWk}\right)}^{2} \end{array}$$ (16)

As in the univariable Radial MR setting, the square root contribution of each instrument to global heterogeneity with respect to an exposure *k* is given by:

$$\begin{array}{c} \sqrt{Q_{kj}}=\beta_{kj}\sqrt{w_{kj}}-\sum_{m=1}^{M}\beta_{m}\sqrt{w_{mj}}-\beta_{IVWk}\sqrt{w_{kj}} \end{array}$$ (17)

Eq (17) describes how the square root contribution to heterogeneity with respect to exposure *k* is represented by the distance from each adjusted point to the superimposed regression line for *β*_*IVWk*_, evaluated at $\sqrt{w_{kj}}$.

**Fig 3:**
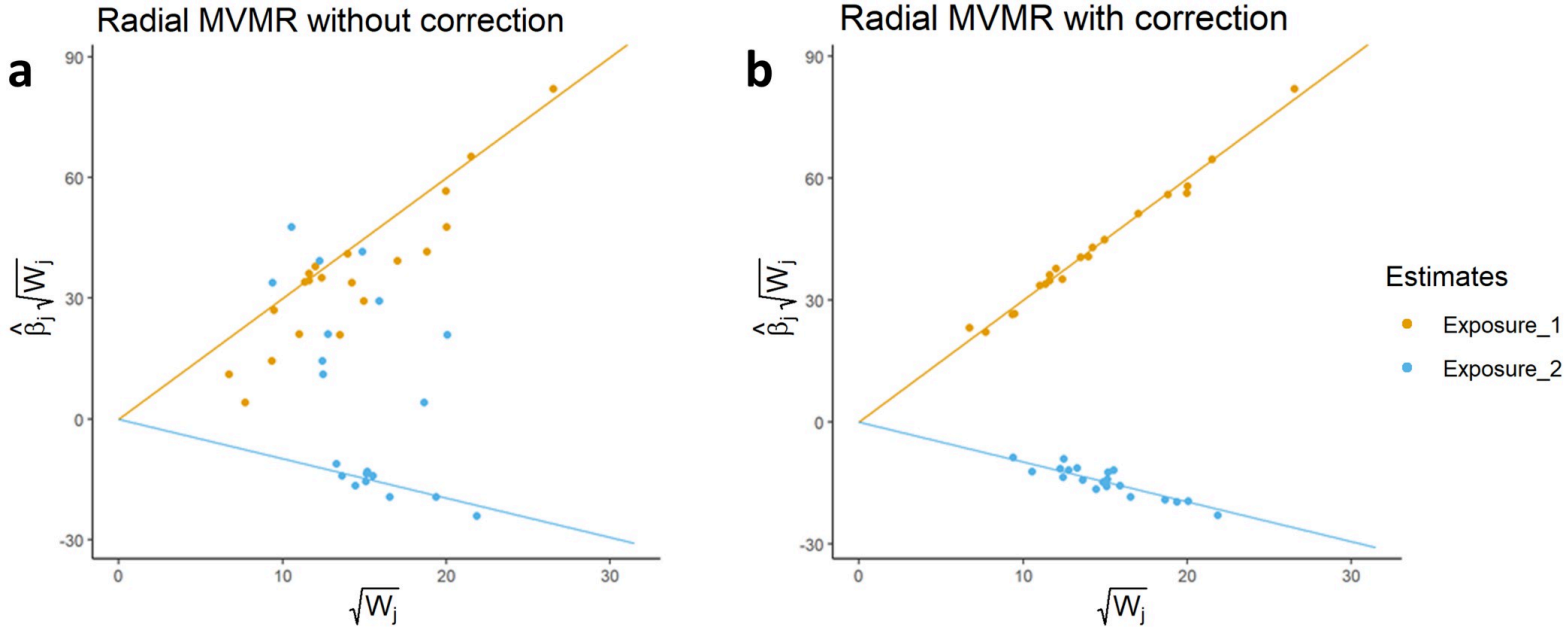
A pair of RMVMR plots using two exposures presented for illustration. In this case, both exposures have a non-zero effect on the outcome, and 10 instruments are associated with both exposures simultaneously. Fig a uses ratio estimates prior to adjusting for the additional exposure, resulting in substantial observed heterogeneity. Fig b shows a substantial reduction in observed heterogeneity when adjusting for the additional exposure.

To visualise the extent to which the addition of an exposure minimises effect estimate heterogeneity, a pair of RMVMR plots can be constructed. Initially an RMVMR plot is created including regression lines showing the MVMR estimate for each exposure. For the first plot, each data point shows the the square root weighting for each instrument $\left(\sqrt{w_{kj}}\right)$, and the product of the square root weighting and unadjusted ratio estimate for each exposure. The second RMVMR plot includes the adjustment to each univariable ratio estimate, described in Eq (15) and therefore accounts for the inclusion of the other exposures in the model. In each case the fitted line is the estimated effect from the MVMR estimation. An example of such plots using simulated data is presented in Fig 3.

In this simulated example a total of 30 instruments are used, of which 10 are associated with exposure *X*_1_, 10 are associated with exposure *X*_2_, and a final 10 instruments are associated with *X*_1_ and *X*_2_ simultaneously. In univariable analyses, provided both exposures have an effect on the given outcome 10 instruments will violate IV3 through the omitted exposure. This is shown by the degree of observed heterogeneity in Fig 3a prior to using adjusted ratio estimates. As shown in Fig 3b, where the inclusion of an additional exposure accounts for remaining pleiotropic effects, the resulting adjusted ratio estimates will converge to the MVMR estimate, that is, the direct effect of each exposure on the outcome of interest.

A number of important features of can be discerned from Fig 3 which warrant consideration. Initially, the degree to which the position of data points changes can serve to indicate whether the inclusion of an additional exposure within an RMVMR model is appropriate. Specifically, if omitting an exposure induces bias when calculating ratio estimates, we would expect the vertical position of data points to change when applying an adjustment. In cases where data points do not appreciably change in position, this can imply either that the additional exposure has no effect on the outcome (*β*_*m*_ = 0), that instruments are exposure specific such that *w*_*mj*_ = 0, or an unlikely scenario where the adjustments across all exposures are balanced such that $\sum_{m=1}^{M}\beta_{m}\sqrt{w_{mj}}=0$.

A second benefit of RMVMR plots is the ability to visually identify the relative exposure-specific weighting of each instrument. In Fig 3 the position of each instrument on the x-axis reflects its weighting ($\sqrt{w_{j}}$) with respect to each individual exposure. It should be noted, however, that this does not reflect the conditional instrument strength of each instrument.

Finally, as highlighted in Eq (17) the vertical distance of each data point in Fig 3b from their corresponding superimposed regression line is equal to their square root contribution to heterogeneity with respect to the given exposure. This can be indicative of invalid instruments, and would warrant further follow-up using external data. It is, however, critical to note that the adjustments made to each exposure are reliant upon initial estimates for the direct effect of each exposure ${\hat{\beta }}_{IVWk}$. In cases where these estimates are initially biased an iterative process can be applied, identifying and removing outliers and repeating effect estimation until no outliers exceeding a given Q-statistic threshold are present. However, as in univariable MR, this is reliant upon such outliers being invalid. In cases where the majority of instruments are pleiotropic with a similar distribution of pleiotropic effects, it is possible that valid instruments will be identified as outliers. In these cases, the removal of outliers can lead to estimates converging towards biased estimates of ${\hat{\beta }}_{IVWk}$.

### The RMVMR R package

The RMVMR R package is a tool designed to facilitate the implementation and visualisation of RMVMR analyses. RMVMR analyses should ideally be performed in five stages. First, summary GWAS data need to be obtained for a set of instruments, including instrument-exposure associations for all included exposures, instrument-outcome associations, and corresponding standard errors. With this complete, the data are formatted for downstream analyses using the format_rmvmr() function, and conditional instrument strength is evaluated using the strength_rmvmr() function. Causal effect estimates and tests for pleiotropic instruments can then be performed using the ivw_rmvmr() and pleiotropy_rmvmr() functions. Finally, the plot_rmvmr() function can be used to construct RMVMR plots.

Outliers are detected based on their contribution to heterogeneity after adjustment, and are calculated with respect to each individual exposure. The significance level for identifying outliers can be defined by the user, and a data frame containing the Q-statistics for each individual variant is provided as an output from the pleiotropy_rmvmr() function. Identified outliers can then be followed up using external sources such as PhenoScanner or the MR Base online software platform [14, 15]. The RMVMR package builds upon the RadialMR and MVMR R packages, and can be used in conjunction with data obtained using the MR Base platform. Further details for the RMVMR software and installation instructions are available at https://github.com/WSpiller/RMVMR.

## Verification and comparison

To demonstrate the implementation and advantages of RMVMR a simulation study is presented comprised of two components (simulations 1 and 2). In simulation 1 a single data frame is generated and analysed. This serves to illustrate how RMVMR analyses are implemented and interpreted in an individual case, including outlier detection and plot construction. In the second simulation we explore the sensitivity of RMVMR to a range of different levels of balanced and unbalanced pleiotropy. All RMVMR analyses are performed using the RMVMR R package.

### Simulation 1: Demonstrating the application of RMVMR using a single data frame

Each data frame is simulated so as to include *N* = 200, 000 observations of *J* = 240 instruments *G*_*j*_, three exposures *X*_1−3_, a single unmeasured confounder *U* and an outcome *Y*. The data were generated using a data generating model conforming to Eqs (5) and (6). The set of instruments were generated so as to represent eight equal groups based on their association with one or more exposures, and were assigned arbitrary identification (rsid) numbers. Simulated groups of instruments include:

- Instruments associated with *X*_1_ only (group 1: rs1–30)
- Instruments associated with *X*_2_ only (group 2: rs31–60)
- Instruments associated with *X*_3_ only (group 3: rs61–90)
- Instruments associated with *X*_1_ and *X*_2_ (group 4: rs91–120)
- Instruments associated with *X*_1_ and *X*_3_ (group 5: rs121–150)
- Instruments associated with *X*_2_ and *X*_3_ (group 6: rs151–180)
- Instruments associated with *X*_1_, *X*_2_ and *X*_3_ (group 7: rs181–210)
- Instruments associated with *X*_1_, *X*_2_ and *X*_3_ with a direct effect on Y (group 8: rs211–240)

Non-zero associations between instruments and exposures were randomly sampled from a normal distribution with mean 0 and standard deviation 10. To ensure strong instruments were used in the analysis, values with an absolute value less than 2 were resampled. The effects of *X*_1_, *X*_2_ and *X*_3_ upon *Y* were defined as *β*_1_ = 1, *β*_2_ = 0.2, and *β*_3_ = −0.5 respectively. Exposures were also simulated so as to be correlated, with a correlation coefficient ranging from −0.5 to 0.5.

Instrument group 8 was subdivided into three equal groups, wherein instruments with a direct effect on *Y* are associated with one of the three exposures *X*_1−3_. The direct effects of instruments in group 8 were sampled from a normal distribution with mean 10 and standard deviation 5, resampling to ensure parameters had an absolute value greater than 2. Finally, each set of instrument-exposure estimates, as well as instrument-outcome associations, were obtained from separate non-overlapping samples.

Initially, univariable radial IVW models are applied using only instruments robustly associated with each exposure (F-statistic >10). Radial MR estimates for each individual exposure are presented in Table 1, while univariable radial plots are provided in Figs A—E in S2 Text.

**Table 1:** Causal effect estimates^1^ obtained using radial MR and RMVMR models with differing exposure combinations^2^.

Model	Estimate (se)	p-value	Q-statistic (p-value)
Univariable Radial IVW			
*X*_1_	1.051 (0.041)	<0.001	9242.04 (< 0.001)
*X*_2_	0.697 (0.069)	<0.001	24012.2 (< 0.001)
*X*_3_	0.342 (0.076)	<0.001	29652.94 (< 0.001)
RMVMR (*X*_1_,*X*_2_)			
*X*_1_	0.971 (0.045)	<0.001	623.81 (< 0.001)
*X*_2_	0.142 (0.046)	0.002	621.41 (< 0.001)
RMVMR (*X*_1_,*X*_2_,*X*_3_)			
*X*_1_	1.098 (0.039)	<0.001	544.08 (< 0.001)
*X*_2_	0.311 (0.041)	<0.001	554.90 (< 0.001)
*X*_3_	-0.422 (0.040)	<0.001	557.61 (< 0.001)
Pruned RMVMR (*X*_1_,*X*_2_,*X*_3_)			
*X*_1_	1.001 (0.010)	<0.001	23.66 (> 0.999)
*X*_2_	0.201 (0.011)	<0.001	26.46 (> 0.999)
*X*_3_	-0.497 (0.011)	<0.001	22.90 (> 0.999)

^1^ True effects of each exposure: *β*_1_ = 1, *β*_2_ = 0.2, *β*_3_ = −0.5.

^2^ Sample size *N* = 200, 000

In Table 1 we can see that effect estimates exhibit substantial bias when estimated using univariable radial IVW. The high Q-statistics estimated for each exposure provide evidence of heterogeneity in estimates obtained using each instrument individually, suggesting potential violations of assumption IV3. When using a two exposure RMVMR model including exposures *X*_1_ and *X*_2_, there continues to be evidence of bias. In this case the observed bias is smaller in magnitude, reflecting how adjustment for both *X*_1_ and *X*_2_ accounts for a proportion of the pleiotropic bias observed in univariable analyses. This is expected given instrument groups 4, 7, and 8 are simultaneously associated with exposures *X*_1_ and *X*_2_. This interpretation is further supported by a notable decrease in observed heterogeneity for each exposure. For reference, the estimated conditional F-statistics for *X*_1_ and *X*_2_ were 111.32 and 112.22 respectively.

The inclusion of exposure *X*_3_ adjusts for associations between instruments and the outcome through *X*_3_ which are not mediated downstream by either *X*_1_ or *X*_2_. This again results in a substantial decrease in heterogeneity, although the continued presence of instruments from group 8 has the effect of inducing pleiotropic bias. The conditional F-statistics for each exposure were 105.94, 98.21, and 103.46 respectively.

**Fig 4:**
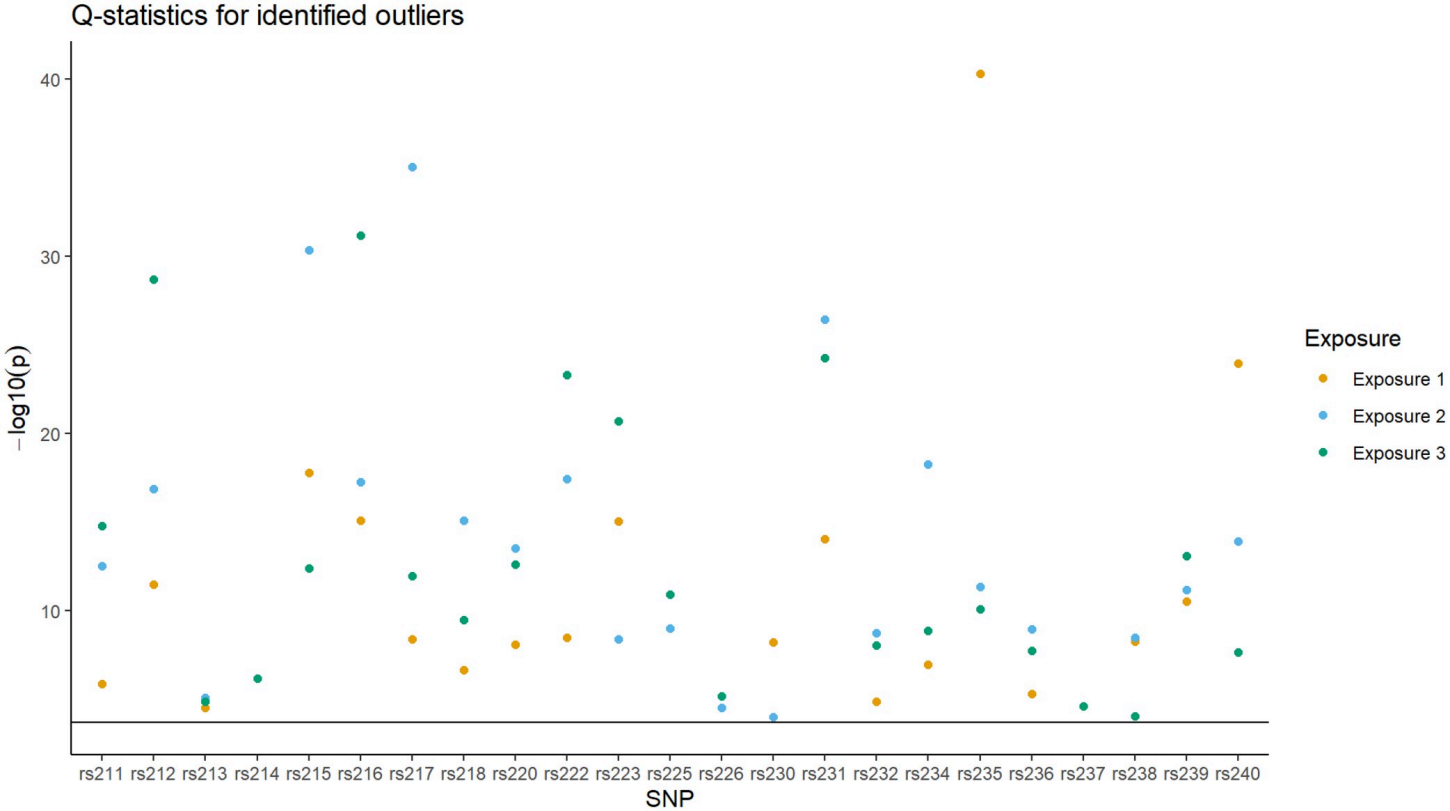
Scatter plot showing instruments which are identified as outliers using the p-value for their contribution to observed heterogeneity. A dotted line is shown representing the p-value threshold for identifying outliers (*p* < 0.05). All instruments correspond to Group 8 (rs211–240) for which a directional pleiotropic effect is present.

By removing instruments identified as outliers on the basis of their contribution to global heterogeneity, it is possible to perform a pruned analysis using the iterative approach previously described. Calculating the individual Q-statistic for each instrument with respect to each exposure, a total of 23 SNPs are identified as outliers using a p-value threshold of 0.05, shown in Fig 4. From Fig 4 it can be seen that all identified outliers correspond to group 8 (rs211–240); instruments generated so as to violate assumption MVMR3 by having a direct effect on the outcome *Y*. Consequently, removing these instruments will have the effect of reducing pleiotropic bias in RMVMR analyses. In Table 1 estimates obtained using the pruned RMVMR approach show no evidence of bias or substantial heterogeneity. The conditional F-statistics for the pruned analysis were 115.56, 107.42, and 114.11 for exposures *X*_1_, *X*_2_, and *X*_3_.

**Fig 5:**
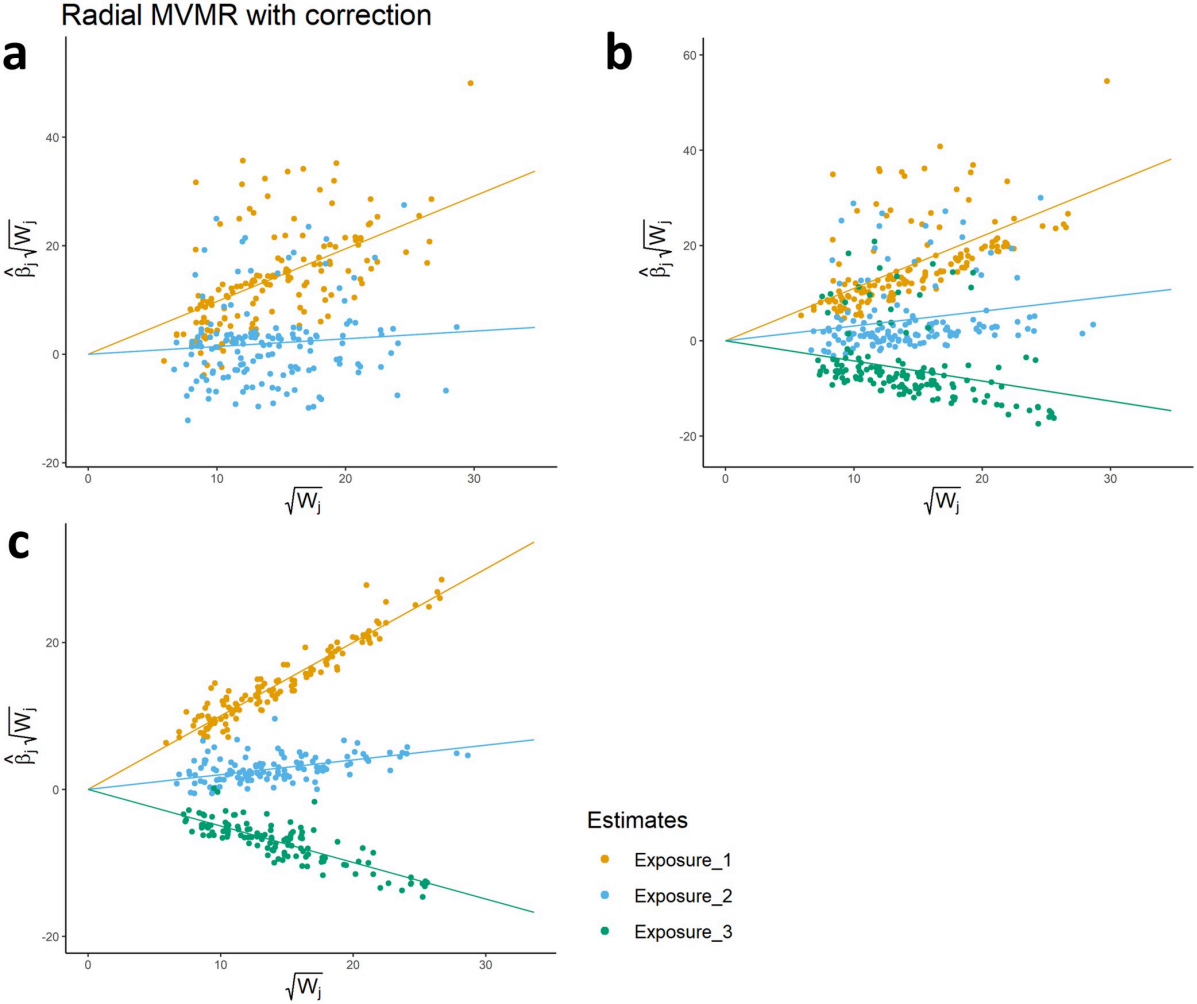
Panel showing radial MVMR plots corresponding to each of the simulated analyses presented in Table 1. Fig a represents the two-exposure model, while Fig b includes all measured exposures. Fig c, shows a plot generated after pruning pleiotropic SNPs identified in Fig 4.

Fig 5 shows RMVMR plots corresponding to the models adopted in simulation 1. In Fig 5s observations do not appear to converge towards their respective effect estimates, instead forming two widely dispersed clusters. The extent to which observations diverge from their corresponding direct effect estimate is representative to their contribution to global heterogeneity, and consequently serves as an indicator of MVMR3 violation.

In Fig 5b the inclusion of exposure *X*_3_ has the effect of substantially reducing global heterogeneity. In this case, a pattern emerges where it is possible to visually identify valid instruments defined in the simulation. The inclusion of instruments from group 8, however, induces pleiotropic bias in causal effects. The continued presence of pleiotropic instruments is indicated by the continued presence of substantial heterogeneity.

Following the systematic pruning of outliers, Fig 5c shows how remaining instruments converge towards unbiased estimates of direct effect for each exposure. Notably, the change of scale on the y-axis reflects the reduction in relative distance from each observation to their respective superimposed regression line, and the absence of substantial heterogeneity suggests an absence of pleiotropic bias. It is, however, important to once again emphasise that such an interpretation is predicated on MVMR3 violating instruments being identified as outliers, and care should be taken when considering outlier removal.

### Simulation 2: Evaluating the performance of Radial MVMR under different levels of pleiotropy

We now consider how sensitive Radial MVMR is to pleiotropy through a set of simulations where the proportion of SNPs with a pleiotropic effect, and whether that pleiotropic effect was balanced or unbalanced, has been varied. In this simulation 210 SNPs were included in the estimation, set up in the same way as groups 1—7 described above. A random selection of these SNPs then had an additional pleiotropic effect on the outcome, the total proportion of SNPs selected to be pleiotropic varied between 0% and 75%. The direction of pleiotropy was either random (balanced pleiotropy) or fixed to position (unbalanced pleiotropy). The mean effect estimate, standard error, Q-statistic and F-statistic for each exposure estimated by Radial MVMR with and without outliers removed are given in Table 2 with balanced pleiotropy and Table 3 for unbalanced pleiotropy. Each simulation has a sample size of 100,000 and 1000 repetitions.

**Table 2:** Causal effect estimates using Radial MVMR with and without outlier removal with varying levels of *balanced* pleiotropy.

% Pleiotropy		*No outlier removal*			*With outliers removed*		
		Estimate	Q-statistic	F-statistic	Estimate	Q-statistic	F-statistic
		(se)	(p-value)		(se)	(p-value)	
*0%*							
	${\hat{\beta }}_{1}$	0.99	25.06	93.59	0.99	22.86	93.62
		(0.010)	(1.00)		(0.010)	(1.00)	
	${\hat{\beta }}_{2}$	0.20	23.46	93.89	0.20	22.64	93.95
		(0.011)	(1.00)		(0.010)	(1.00)	
	${\hat{\beta }}_{3}$	-0.49	23.67	93.69	-0.49	22.75	93.75
		(0.011)	(1.00)		(0.010)	(1.00)	
*10%*							
	${\hat{\beta }}_{1}$	0.99	655.31	93.76	0.99	22.11	92.68
		(0.060)	(0.001)		(0.023)	(1.00)	
	${\hat{\beta }}_{2}$	0.20	665.87	93.62	0.20	22.23	92.96
		(0.063)	0.001		(0.023)	(1.00)	
	${\hat{\beta }}_{3}$	-0.49	654.53	93.83	-0.49	22.07	93.06
		(0.064)	0.001		(0.023)	(1.00)	
*25%*							
	${\hat{\beta }}_{1}$	0.99	1306.09	93.76	0.99	22.64	91.74
		(0.100)	(<0.001)		(0.039)	(1.00)	
	${\hat{\beta }}_{2}$	0.20	1309.03	93.88	0.20	22.51	91.54
		(0.100)	(<0.001)		(0.040)	(1.00)	
	${\hat{\beta }}_{3}$	-0.50	1313.92	93.94	-0.49	22.25	91.93
		(0.100)	(<0.001)		(0.039)	(1.00)	
*50%*							
	${\hat{\beta }}_{1}$	1.00	2058.55	94.04	0.99	23.60	90.53
		(0.141)	(<0.001)		(0.070)	(1.00)	
	${\hat{\beta }}_{2}$	0.20	2045.93	93.84	0.20	23.67	90.07
		(0.141)	(<0.001)		(0.071)	(1.00)	
	${\hat{\beta }}_{3}$	-0.50	2058.95	93.94	-0.49	23.54	89.74
		(0.141)	(<0.001)		(0.071)	(1.00)	
*75%*							
	${\hat{\beta }}_{1}$	0.99	2609.23	93.82	0.99	24.95	88.71
		(0.172)	(<0.001)		(0.117)	(0.912)	
	${\hat{\beta }}_{2}$	0.21	2619.95	94.02	0.20	24.86	89.13
		(0.173)	(<0.001)		(0.118)	(0.913)	
	${\hat{\beta }}_{3}$	-0.49	2634.33	93.83	-0.49	24.88	88.70
		(0.173)	(<0.001)		(0.117)	(0.908)	

True effects of each exposure: *β*_1_ = 1, *β*_2_ = 0.2, *β*_3_ = −0.5. Sample size *N* = 200, 000

**Table 3:** Causal effect estimates using Radial MVMR with and without outlier removal with varying levels of *unbalanced* pleiotropy.

% Pleiotropy		*No outlier removal*			*With outliers removed*		
		Estimate	Q-statistic	F-statistic	Estimate	Q-statistic	F-statistic
		(se)	(p-value)		(se)	(p-value)	
*0%*							
	${\hat{\beta }}_{1}$	0.99	24.06	93.84	0.99	22.91	93.93
		(0.010)	(1.00)		(0.010)	(1.00)	
	${\hat{\beta }}_{2}$	0.20	23.47	93.65	0.20	22.64	93.71
		(0.011)	(1.00)		(0.010)	(1.00)	
	${\hat{\beta }}_{3}$	-0.49	23.67	93.40	-0.49	22.75	93.48
		(0.011)	(1.00)		(0.010)	(1.00)	
*10%*							
	${\hat{\beta }}_{1}$	1.06	631.62	93.69	1.00	21.08	91.83
		(0.063)	(0.001)		(0.024)	(1.00)	
	${\hat{\beta }}_{2}$	0.27	621.11	93.75	0.21	21.16	92.09
		(0.063)	0.001		(0.024)	(1.00)	
	${\hat{\beta }}_{3}$	-0.42	633.61	93.69	-0.49	21.11	91.23
		(0.063)	0.001		(0.024)	(1.00)	
*25%*							
	${\hat{\beta }}_{1}$	1.18	1116.94	93.83	1.023	20.06	85.21
		(0.096)	(<0.001)		(0.046)	(1.00)	
	${\hat{\beta }}_{2}$	0.38	1117.88	93.65	0.24	19.92	85.51
		(0.096)	(<0.001)		(0.046)	(1.00)	
	${\hat{\beta }}_{3}$	-0.31	1124.72	93.42	-0.45	20.31	84.98
		(0.096)	(<0.001)		(0.047)	(1.00)	
*50%*							
	${\hat{\beta }}_{1}$	1.34	1528.46	93.83	1.19	25.79	82.72
		(0.127)	(<0.001)		(0.092)	(0.867)	
	${\hat{\beta }}_{2}$	0.56	1547.58	93.70	0.40	25.81	81.48
		(0.127)	(<0.001)		(0.094)	(0.858)	
	${\hat{\beta }}_{3}$	-0.15	1526.46	93.28	-0.30	25.85	81.78
		(0.127)	(<0.001)		(0.093)	(0.873)	
*75%*							
	${\hat{\beta }}_{1}$	1.52	1704.81	93.65	1.45	32.58	85.92
		(0.146)	(<0.001)		(0.124)	(0.573)	
	${\hat{\beta }}_{2}$	0.72	1710.06	93.67	0.650	32.21	86.27
		(0.146)	(<0.001)		(0.125)	(0.573)	
	${\hat{\beta }}_{3}$	0.038	1725.78	93.83	-0.03	32.60	86.07
		(0.146)	(<0.001)		(0.125)	(0.575)	

True effects of each exposure: *β*_1_ = 1, *β*_2_ = 0.2, *β*_3_ = −0.5. Sample size *N* = 200, 000

When the pleiotropic effects of the SNPs are balanced to have both positive and negative effects on the estimates obtained we see that increasing the proportion of pleiotropy does not bias the effect estimate obtained. However, the uncertainty in that estimate increases substantially, leading to large Q-statistics. Removing the outliers in this setting lowers the uncertainty around the effect estimate obtained. The standard errors in the estimation with outliers removed remain larger than in the setting with no pleiotropy, partly because the number of SNPs included in the pruned analysis will be lower as the the proportion of SNPs that are outlying increases.

When the pleiotropic effects of the SNPs are unbalanced the bias of the effect estimates obtained increases with the proportion of the SNPs that are pleiotropic. Removing outliers in the model decreases the bias and for moderate levels of pleiotropy gives effect estimates that are very close to the true values. When the proportion of pleiotropic SNPs is high (≥ 50%) there is still substantial bias in the effect estimates after outlier removal. This occurs because the assumption that all of the pleiotropic SNPs will be identified as outliers is no longer satisfied.

Overall these results highlight how Radial MVMR with outlier removal can be used to obtain more reliable effect estimates under moderate levels of pleiotropy but will not recover unbiased effect estimates under high levels of directional pleiotropy.

## Application

### Estimation of the effect of lipid fractions on coronary heart disease

To illustrate the RMVMR approach in an applied setting we consider the effects of lipid fractions, specifically high-density lipoprotein cholesterol (HDL), low-density lipoprotein cholesterol LDL, and triglycerides, on coronary heart disease (CHD). SNP-exposure estimates were obtained from previously published GWAS summary data, using data from the Global Lipids Genetics Consortium presented in Willer et al [16]. Each lipid fraction was recorded in mg/Dl, and standardised before GWAS were performed. SNP-outcome associations were obtained from the CARDIoGRAMplusC4D Consortium as presented in Nikpay et al [17]. CHD associations are presented on a log-odds scale, and were obtained using logistic regression. All SNP associations were obtained using the MRBase online platform [14]. For each exposure, univariable radial MR analyses are performed, after which radial MVMR models including all exposures are fit to the data.

We identified SNPs strongly associated with each exposure (*p* < 5 × 10^−8^). Independent SNPs were retained using a linkage disequilibrium clumping threshold of *R*^2^ < 0.001, and palindromic SNPs were also removed prior to performing analyses. This resulted in a total of 87 SNPs associated with HDL, 67 SNPs associated with LDL, and 40 SNPs associated triglycerides being selected for subsequent analyses. These SNPs were extracted from the GWAS of CHD and were harmonised to ensure consistent effect alleles. For each exposure we calculated the univariable radial MR effect estimates, heterogeneity Q statistics and mean F-statistic.

We then combined the list of SNPs for each exposure and performed an additional round of clumping to remove any SNPs for one exposure that were in LD with a SNP for another exposure. This gave a resulting list of 153 SNPs as instruments for our MVMR analysis. For each of these SNPs we then extracted SNP-trait associations for each of HDL, LDL and Triglycerides. We used these to estimate the direct effect of each exposure in a single model with radial MVMR, estimate exposure specific heterogeneity statistics and conditional F-statistics to test instrument strength. We finally applied pruning to remove any SNPs that contributed more to the heterogeneity statistics than would be expected by chance and re-estimated the radial MVMR model without them included. Results from all of the analyses conducted are presented in Table 4.

**Table 4:** Causal effect estimates obtained using radial MR and radial MVMR models, estimating the effect of lipid fractions (HDL, LDL, and triglycerides) on CHD.

Model	Estimate	Std. Error	P-value	Q-statistic	F-Statistic
	(Odds Ratio)			(p-value)	
Univariable Radial IVW					
HDL^1^	-0.188	0.058	0.001	444.90	121.6
	(0.829)			(< 0.001)	
LDL^1^	0.405	0.055	< 0.001	229.35	100.1
	(1.499)			(< 0.001)	
Triglycerides^1^	0.227	0.062	< 0.001	162.81	170.3
	(1.255)			(< 0.001)	
RMVMR					
HDL	-0.106	0.053	0.046	144.24^2^	42.9
	(0.899)			(0.004)	
LDL	0.392	0.057	< 0.001	109.36^2^	39.2
	(1.408)			(0.020)	
Triglycerides	0.116	0.064	0.074	84.13^2^	29.1
	(1.123)			(0.055)	
Pruned RMVMR					
HDL	-0.062	0.042	0.135	74.39^2^	43.6
	(0.939)			(0.910)	
LDL	0.335	0.050	< 0.001	40.13^2^	32.2
	(1.399)			(0.997)	
Triglycerides	0.136	0.055	0.014	46.16^2^	24.9
	(1.146)			(0.869)	

As the outcome GWAS summary statistics were only available as log odds ratios estimated effect estimates are given in log odds ratio (Estimate) and converted into odds ratios (given in parenteses). Standard errors given based on the log odds ratio.

^1^ Estimates obtained using univariable radial MR.

^2^ Calculated using corrected ratio estimates

The results given in Table 4 highlight how failure to account for the other exposures and potential pleiotropy can give potentially misleading results. Each of the univariable Radial results are attenuated towards the null once the other exposures are accounted for in the Radial MVMR estimation. Radial MVMR estimates for each exposure are shown in Table 4, showing the effect of each lipid fraction to be directionally consistent, though smaller in magnitude, compared to univariable estimates. Assuming that the observed heterogeneity in univariable analyses is the result of omitting one or more lipid fractions, performing a correction for each observation and re-evaluating heterogeneity using radial MVMR should show evidence of a substantial Q-statistic decrease. Table 4 shows a substantial decrease in observed heterogeneity when fitting the radial MVMR model, though HDL and LDL still show evidence of significant global heterogeneity using a Q-statistic p-value threshold of *p* < 0.05.

When SNPs that contributed more to the heterogeneity statstic than would be expected by chance were removed effect estimates remain directionally consistent with the initial radial MVMR analysis. The effect estimates obtained were of smaller magnitude for HDL and LDL, and the effects of all exposures are estimated with greater precision. Importantly, the conditional F-statistic for each exposure remains at a similar level to the initial radial MVMR analysis, limiting the extent to which differences in estimation are the result of bias due to weak instruments being used after pruning.

Removing SNPs which exhibit heterogeneity does not necessarily imply that estimates will be less biased. If the majority of SNPs exhibit pleiotropic effects in a similar direction and magnitude, it is possible that SNPs satisfying the MVMR assumptions will be removed. To consider this possibility it is important to follow-up identified outliers using external data, focusing on associations with phenotypes for which a pleiotropic association is plausible. In the pruned analysis, a total of 17 SNPs were identified and removed as outliers (see S2 Text). Using the PhenoScanner online platform to evaluate potential pleiotropic pathways, there did not appear to be a consistent pattern across the set of removed SNPs, though phenotypes such as diastolic blood pressure are present [15].

**Fig 6:**
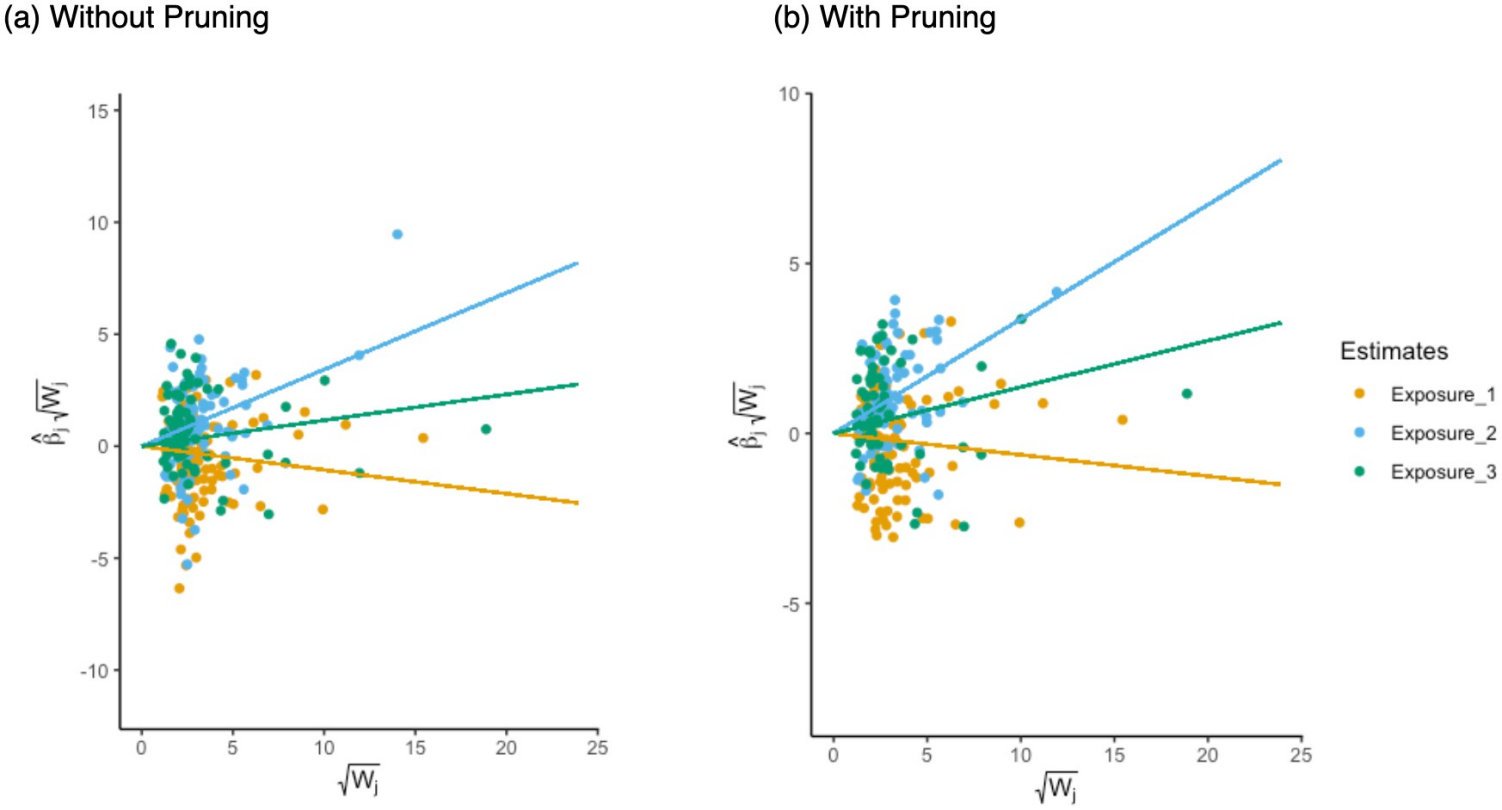
Panel showing RMVMR plots for applied analysis using HDL, LDL, and triglycerides. Exposure 1—HDL Cholesterol, Exposure 2—LDL Cholesterol, Exposure 3—Triglycerides. Observations correspond to ratio estimates and weightings with respect to each exposure. Regression lines represent MVMR causal effect estimates coloured by exposure. Fig a shows the radial MVMR estimates prior to performing heterogeneity pruning, while Fig b shows the radial MVMR estimates using heterogeneity pruned summary data.

The radial MVMR estimates are visualised in Fig 6, and adjusted Q-statistics are presented in Table 4. The reduction in observed heterogeneity suggests that univariable analyses exhibit bias when failing to account for pleiotropic associations through other lipid fractions. A multivariable model would therefore appear to be a more effective approach in this instance. The plot shown in Fig 6a show the estimates obtained without pruning SNPs based on their heterogeneity contribution, while plot Fig 6b shows the plots constructed after removing observed outliers. In this case, the reduction in global heterogeneity is clear and primarily reflected by the change of scale on the y-axis, in combination with the data points for each exposure being substantially closer to their corresponding superimposed regression lines.

When pruning for outliers the effect of HDL is greatly attenuated, showing no evidence of an effect on CHD. LDL and triglycerides continue to show evidence of a positive association, with LDL having the most substantial impact on CHD risk. Provided a majority of valid SNPs with respect to their weighting have been used, this would suggest that LDL and triglycerides represent promising targets for CHD prevention.

## Discussion

Radial MR and MVMR approaches facilitate the assessment of pleiotropic associations between genetic variants and phenotypes using GWAS summary data. Conducting analyses within a radial framework allows for outliers to be effectively visualised, and MVMR analyses allow for the direct effects of multiple exposures to be estimated simultaneously. Radial MVMR builds upon both these existing methods, providing a means for visualising MVMR approaches absent until this point, and an approach of outlier identification and removal in a MVMR framework. We propose that the radial MVMR approach be used to assist in communicating key findings as a visual aid, and also as a sensitivity analysis for identifying pleiotropic bias using adjusted heterogeneity statistics.

When implementing the Radial MVMR approach it is crucial to consider the underlying assumptions of MVMR. Instruments selected should be sufficiently strong so as to overcome substantial weak instrument bias, estimated using the conditional F-statistic. Specific to Radial MVMR, the correction of individual ratio estimates is reliant upon unbiased estimates of the direct effect of each exposure. As demonstrated in the simulation study, if the majority of the SNPs have pleiotropic effects in the same direction the outlier adjusted Radial MVMR estimates will still be biased.

As previously emphasised, care should be taken to consider identified outliers and phenotypic associations which could plausibly form horizontal pleiotropic pathways. If a majority of instruments have direct effects upon an outcome, and the distribution of such direct effects is similar, it is likely that SNPs satisfying the MVMR assumptions will be identified as outliers. As a consequence, the removal of such SNPs would result in estimates converging toward the biased estimate produced by such pleiotropic SNPs. Heterogeneity in per SNP effect estimates can arise due to mechanisms other than horizontal pleiotropy, for example tissue specificity in the SNP effects will lead to heterogeneity when the samples are taken from a single tissue. [18] Decisions to down weight or remove outliers during an analysis should be made with consideration of the biological mechanisms underlying observed SNP-phenotype associations, and adequate justification. It is with this in mind that a general heterogeneity pruning function has not been incorporated within the RMVMR R package, though code for performing such analyses is available at https://github.com/WSpiller/RMVMR_Analyses.

A further issue related to the applied analysis is the use of binary outcomes in summary MR analyses. When using SNP-outcome associations estimated on a log-odds scale, it is possible that causal estimates will be correlated with their precision, introducing heterogeneity which is not a consequence of pleiotropic associations [7, 19]. This issue, which is a wider issue within the summary MR literature, warrants careful consideration prior to performing analyses, and care should be taken in evaluating as an indicator of pleiotropy when a binary outcome is used [19].

Finally, it should be noted that while first-order weights have been used throughout this paper, radial approaches allow for a wide-range of weighting options to be used. As arbitrary weights can be used, should be possible for modified second order weights to be incorporated with a radial MVMR model. Such weights may prove more effective than first-order weights, as they incorporate the precision of SNP-exposure estimates mitigating violations of the NO-Measurement Error (NOME) assumption in summary MR [20]. Future work will explore how differing weight specifications can improve estimation using radial MVMR.

## Supporting information

S1 Text Derivation of the Radial MVMR ratio estimate. (DOCX)

S2 Text Additional plots from our simulation study. **Fig A.** A Radial MR plot showing the estimated causal effect of exposure *X*_1_ upon outcome Y. Observations represent the ratio estimate for each SNP robustly associated with exposure *X*_1_. **Fig B.** A Radial MR plot showing the estimated causal effect of exposure *X*_2_ upon outcome Y. Observations represent the ratio estimate for each SNP robustly associated with exposure *X*_2_. **Fig C.** A Radial MR plot showing the estimated causal effect of exposure *X*_3_ upon outcome Y. Observations represent the ratio estimate for each SNP robustly associated with exposure *X*_3_. **Fig D.** A Radial MR plot showing heterogeneity indicative of conditional instrument strength for exposure *X*_1_. **Fig E.** A Radial MR plot showing heterogeneity indicative of conditional instrument strength for exposure *X*_2_.
(DOCX)
