# Supplementary material for: Estimating and visualising multivariable Mendelian randomization analyses within a radial framework
Source: PLoS Genet. 2024 Dec 16;20(12):e1011506. doi: 10.1371/journal.pgen.1011506 (PMC11684766; doi:10.1371/journal.pgen.1011506)
Supplement: S1 Text — (DOCX) [file pgen.1011506.s001.docx]

**Estimating and visualising multivariable Mendelian randomization analyses within a radial framework.**

**Supplementary Text 1**

Wes Spiller^1,2^, Jack Bowden^3,4^ and Eleanor Sanderson^1,2^

1. Population Health Sciences, University of Bristol, Bristol, UK
2. MRC Integrative Epidemiology Unit, University of Bristol, Bristol, UK
3. University of Exeter Medical School, Exeter, UK
4. Novo Nordisk Genetics Centre of Excellence, Oxford

# Derivation of the ratio estimate

For $i\in\left( 1,2,\ldots,N \right)$ observations including $j\in(1,2,\ldots,J)$ SNPs, a set of SNPs $l\in\left( 1,2,\ldots L \right)$ where $j\notin l$, $k\in\left( 1,2,\ldots,K \right)$ exposures, and a set of exposures $m\in\left( 1,2,\ldots,m \right)$ for which $k\notin m$:

Data Generating Model

$G_{ji}\sim\theta_{j0}+\sum_{l=1}^{L} \theta_{jl}G_{l}+\epsilon_{Gji}$ (A2.1)

$U_{i}=\pi_{0}+\sum_{j=1}^{J} \pi_{j}G_{j}+\epsilon_{U_{i}}$ (A2.2)

$X_{ki}=\gamma_{k0}+\sum_{j=1}^{J} \gamma_{kj}G_{j}+\gamma_{k\left( J+1 \right)}U_{i}+\sum_{m=1}^{M} \delta_{km}X_{m}+\epsilon_{Xki}$ (A2.3)

$Y_{i}=\beta_{0}+\sum_{k=1}^{K} \beta_{k}X_{k}+\sum_{j=1}^{J} \alpha_{j}G_{j}+\beta_{\left( K+1 \right)}U_{i}+\epsilon_{Yi}$ (A2.4)

Linear regression of $Y$ on all variables

$Y_{i}=\hat{\beta}_{0}+\sum_{k=1}^{K} \hat{\beta}_{k}X_{k}+\sum_{j=1}^{J} \hat{\alpha}_{j}G_{j}+\hat{\beta}_{\left( K+1 \right)}U_{i}+\hat{\epsilon}_{Yi}$ (A2.5)

Simple linear regression of $G_{j}$ on $G_{l}$

$G_{ji}=\tilde{\theta}_{lj0}+{\tilde{\theta}_{jl}G}_{li}+\tilde{\epsilon}_{Gjli}$ (A2.6)

Simple linear regression of $U$ on $G_{j}$

$U_{i}=\tilde{\pi}_{j0}+\tilde{\pi}_{j}G_{j}+\tilde{\epsilon}_{Uji}$ (A2.7)

Linear regression of $Y$ on all variables

$X_{ki}=\hat{\gamma}_{k0}+\sum_{j=1}^{J} \hat{\gamma}_{kj}G_{j}+\hat{\gamma}_{k\left( J+1 \right)}U_{i}+\epsilon_{Xki}$ (A2.8)

Simple linear regression of $X_{k}$ on $G_{j}$

$X_{k}=\tilde{\gamma}_{kj0}+\tilde{\gamma}_{kj}G_{j}+\tilde{\epsilon}_{Xkji}$ (A2.9)

Total effect of $G_{j}$ on exposure $X_{k}$

$\tilde{\gamma}_{kj}=\hat{\gamma}_{kj}+\sum_{l=1}^{L} \hat{\gamma}_{kl}\hat{\theta}_{jl}+\sum_{m=1}^{M} \delta_{km}\tilde{\gamma}_{kj}+\gamma_{k\left( J+1 \right)}\tilde{\pi}_{j}$ (A2.9)

Reduced form simple linear regression of Y on instrument $G_{j}$

$Y_{i}=\tilde{\Gamma}_{j0}+\tilde{\Gamma}_{ji}+\tilde{\eta}_{i}$ (A2.10)

Total effect of $G_{j}$ on $Y$

$\tilde{\Gamma}_{j}=\sum_{k=1}^{K} \hat{\beta}_{k}\tilde{\gamma}_{kj}+\hat{\alpha}_{j}+\sum_{l=1}^{L} \hat{\alpha}_{l}\tilde{\theta}_{lj}+\hat{\beta}_{K+1}\tilde{\pi}_{j}$ (A2.11)

Wald ratio estimate $\hat{\beta}_{kj}$ for instrument $G_{j}$

$\hat{\beta}_{kj}=\frac{\tilde{\Gamma}_{j}}{\tilde{\gamma}_{kj}}=\frac{\sum_{k=1}^{K} \hat{\beta}_{k}\tilde{\gamma}_{kj}+\hat{\alpha}_{j}+\sum_{l=1}^{L} \hat{\alpha}_{l}\tilde{\theta}_{lj}+\hat{\beta}_{K+1}\tilde{\pi}_{j}}{\hat{\gamma}_{kj}+\sum_{l=1}^{L} \hat{\gamma}_{kl}\hat{\theta}_{jl}+\sum_{m=1}^{M} \delta_{km}\tilde{\gamma}_{mj}+\gamma_{k\left( J+1 \right)}\tilde{\pi}_{j}}$ (A2.12)

$\frac{\tilde{\Gamma}_{j}}{\tilde{\gamma}_{kj}}=\hat{\beta}_{k}+\frac{\sum_{m=1}^{M} \hat{\beta}_{m}\tilde{\gamma}_{mj}+\hat{\alpha}_{j}+\sum_{l=1}^{L} \hat{\alpha}_{l}\tilde{\theta}_{lj}+\hat{\beta}_{K+1}\tilde{\pi}_{j}}{\hat{\gamma}_{kj}+\sum_{l=1}^{L} \hat{\gamma}_{kl}\hat{\theta}_{jl}+\sum_{m=1}^{M} \delta_{km}\tilde{\gamma}_{mj}+\gamma_{k\left( J+1 \right)}\tilde{\pi}_{j}}$ (A2.13)

MVMR1: $\tilde{\gamma}_{kj}=\hat{\gamma}_{kj}+\sum_{l=1}^{L} \hat{\gamma}_{kl}\hat{\theta}_{jl}+\sum_{m=1}^{M} \delta_{km}\tilde{\gamma}_{mj}\neq0$

MVMR2: $\pi_{j}=0$

MVMR3: $\alpha_{j}=0$

$\frac{\tilde{\Gamma}_{j}}{\tilde{\gamma}_{kj}}=\hat{\beta}_{k}+bias\left( \frac{\sum_{m=1}^{M} \hat{\beta}_{m}\tilde{\gamma}_{mj}}{\hat{\gamma}_{kj}+\sum_{l=1}^{L} \hat{\gamma}_{kl}\hat{\theta}_{jl}+\sum_{m=1}^{M} \delta_{km}\tilde{\gamma}_{mj}} \right)$ (A2.14)

$\frac{\tilde{\Gamma}_{j}}{\tilde{\gamma}_{kj}}=\hat{\beta}_{k}+bias\left( \frac{\sum_{m=1}^{M} \hat{\beta}_{m}\tilde{\gamma}_{mj}}{\tilde{\gamma}_{kj}} \right)$ (A2.15)

When the MVMR assumptions are satisfied, the univariable ratio bias term using a single instrument with respect to exposure $X_{k}$ is equal to the sum of the additional effects of exposures $X_{m}$ divided by the total effect of the SNP on exposure $X_{k}$. This is adjusted for when including information on exposures $X_{m}$ within an MVMR model.
