# Supplementary material for: Estimating and visualising multivariable Mendelian randomization analyses within a radial framework
Source: PLoS Genet. 2024 Dec 16;20(12):e1011506. doi: 10.1371/journal.pgen.1011506 (PMC11684766; doi:10.1371/journal.pgen.1011506)
Supplement: S2 Text — Fig A. A Radial MR plot showing the estimated causal effect of exposure X1 upon outcome Y. Observations represent the ratio estimate for each SNP robustly associated with exposure X1. Fig B. A Radial MR plot showing the estimated causal effect of exposure X2 upon outcome Y. Observations represent the ratio estimate for each SNP robustly associated with exposure X2. Fig C. A Radial MR plot showing the estimated causal effect of exposure X3 upon outcome Y. Observations represent the ratio estimate for each SNP robustly associated with exposure X3. Fig D. A Radial MR plot showing heterogeneity indicative of conditional instrument strength for exposure X1. Fig E. A Radial MR plot showing heterogeneity indicative of conditional instrument strength for exposure X2. (DOCX) [file pgen.1011506.s002.docx]

**Estimating and visualising multivariable Mendelian randomization analyses within a radial framework.**

**Supplementary Text 2**

Wes Spiller^1,2^, Jack Bowden^3,4^ and Eleanor Sanderson^1,2^

1. Population Health Sciences, University of Bristol, Bristol, UK
2. MRC Integrative Epidemiology Unit, University of Bristol, Bristol, UK
3. University of Exeter Medical School, Exeter, UK
4. Novo Nordisk Genetics Centre of Excellence, Oxford

# Simulation study: Further information

Univariable Radial MR Plots


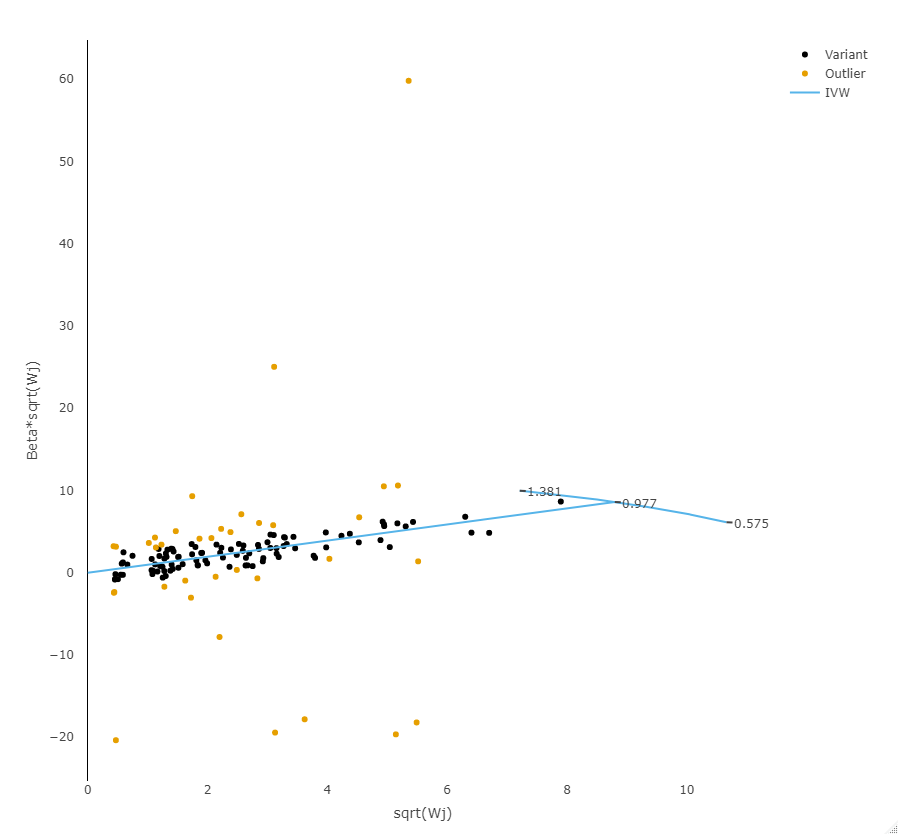


***Supplementary Figure A***: A Radial MR plot showing the estimated causal effect of exposure $X_{1}$ upon outcome $Y$. Observations represent the ratio estimate for each SNP robustly associated with exposure $X_{1}$.


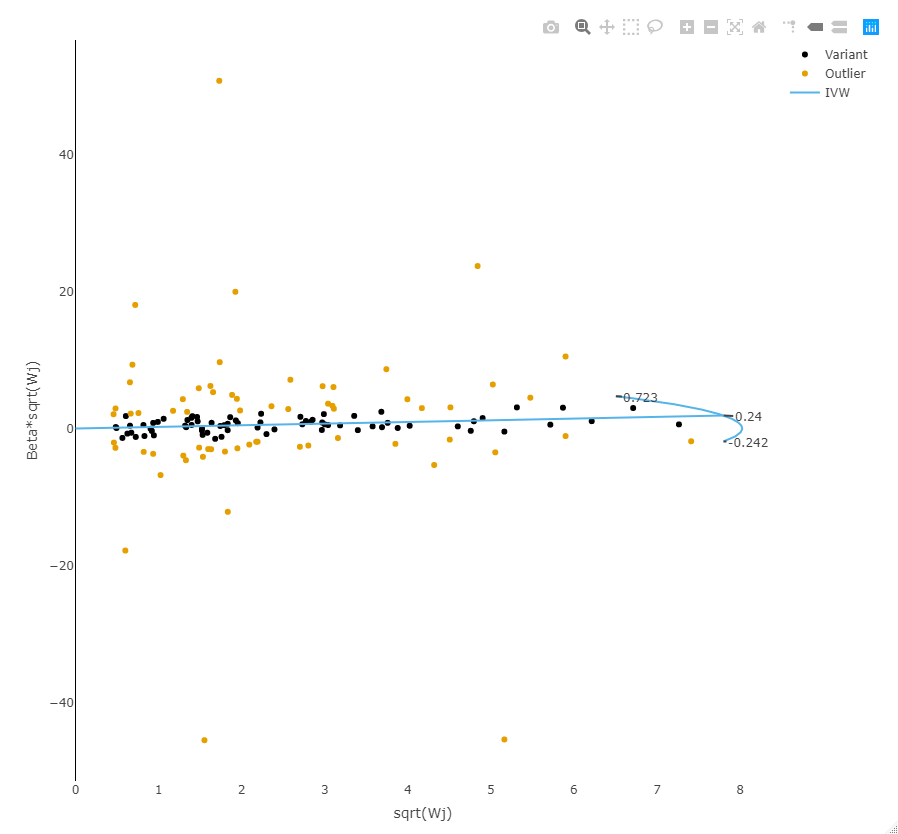


***Supplementary Figure B***: A Radial MR plot showing the estimated causal effect of exposure $X_{2}$ upon outcome $Y$. Observations represent the ratio estimate for each SNP robustly associated with exposure $X_{2}$.


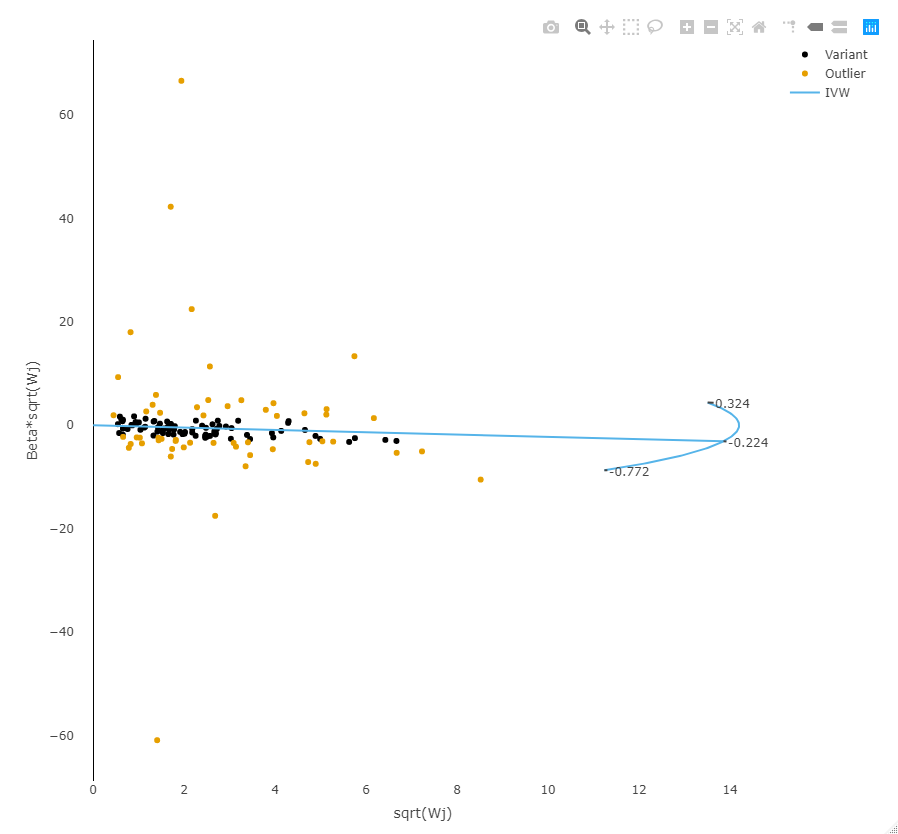


***Supplementary Figure C:***  A Radial MR plot showing the estimated causal effect of exposure $X_{3}$ upon outcome $Y$. Observations represent the ratio estimate for each SNP robustly associated with exposure $X_{3}$.


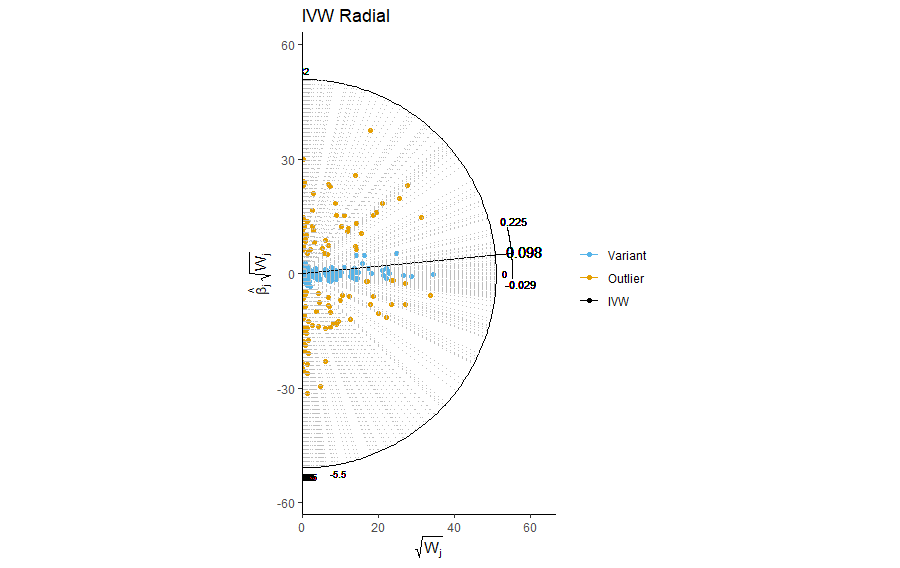


***Supplementary Figure D***: A Radial MR plot showing heterogeneity indicative of conditional instrument strength for exposure $X_{1}$


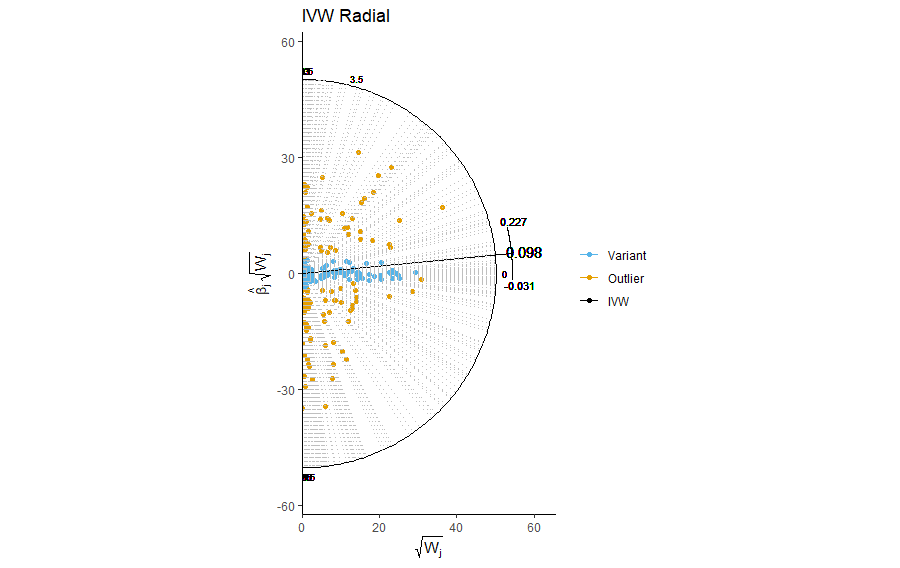


***Supplementary Figure E***: A Radial MR plot showing heterogeneity indicative of conditional instrument strength for exposure $X_{2}$

Additional information on applied example

SNPs identified as outliers in radial MVMR analysis

rs11065987

rs11153594

rs11244084

rs1250229

rs1260326

rs12740374

rs12801636

rs205262

rs2068888

rs2288912

rs2390536

rs267733

rs3106166

rs35633988

rs4530754

rs6567160

rs998584
